# Occurrence of multiclass pesticide residues in tomato samples collected from different markets of Iran

**DOI:** 10.1007/s40201-018-0296-4

**Published:** 2018-05-07

**Authors:** Jamshid Salamzadeh, Attaollah Shakoori, Vahideh Moradi

**Affiliations:** 1 Food Safety Research Center, Shahid Beheshti University of Medical Sciences, Tehran, Iran; 2 Department of Clinical Pharmacy, Shahid Beheshti University of Medical Sciences, Tehran, Iran; 3 Vice-Chancellor for Food and Drug Affairs, Shahid Beheshti University of Medical Sciences, Tehran, Iran; 4 Department of Food Science and Technology, Science and Research Branch, Islamic Azad University, Tehran, Iran

**Keywords:** Pesticide residues, Selected ion monitoring, GC-MS, Iran, Tomato

## Abstract

**Background:**

Pesticides are a reason for popular concern due to their possible unfavorable results on human safety. Most pesticide residues are present in food owing to the direct application of a pesticide to a crop. The aims of this study were; development a multiresidue method for analysis of 81 pesticides in tomato using GC/MS, and detection and quantitation of the studied pesticides in tomato samples gathered from various stores of Iran.

**Methods:**

The pesticides were assessed concurrently in a single run applying GC/MS after extraction with QuEChERS method. Homogenized tomato samples were weighed into centrifuge tubes. The studied pesticides were extracted using acetonitrile, followed by the addition of a mixture of anhydrous magnesium sulfate and sodium acetate. In order to remove excess water and other components of tomato a combination of primary secondary amine and magnesium sulfate was applied, and then the extracted components were analyzed by GC-MS.

**Results:**

The calibration curves for all analytes were linear in the range of 20–200 ng/g with a determination coefficient (R^2^) in the range between 0.993 and 0.999. The LODs and LOQs were in the range between 2.5–6.7 and 7.5–20 ng/g respectively, and the mean recoveries obtained for three fortification levels (25,50 and 100 ng/g -five replicates each) were 72–116% with RSD < 20%. Six residues were found in 31 (20.7%) samples. Iprodione was the most common detected residues (6.0%), followed by permethrine (4.7%), esfenvalerate (4.7%), chlorpyrifos (3.3%), diazinon (2.0%), and penconazole (1.3%).

**Conclusions:**

Among the detected pesticides, only Iprodione, permethrine, chlorpyrifos and diazinon are registered for tomato production in Iran. With exception of Chlorpyrifos and diazinon the concentrations of iprodione and permethrine were found below the maximum residue levels (MRLs) established by Iranian National Standard Organization (INSO). Esfenvalerate and penconazole are not registered for tomato production in Iran. Therefore, it is necessary to control and management of their residues in tomato.

## Background

Pesticides consist of a large number of chemicals that are utilized to prevent, destroy, repel, attract, or reduce pest organisms at different stages of cultivation. Up to now, at least 1000 chemicals have been synthesized as active pesticide ingredients in the world and are produced in various formulations by manufacturer. Metabolism and environmental degradation are two major routes that convert pesticides to the different metabolites. Pesticides and their metabolites display very large differences in chemical structure and physical properties [1]. Chemically, they are completely heterogeneous such as organochlorines, carbamates, pyrethroids and substituted ureas [2]. Despite the remarkable economic and agricultural benefits of pesticides, they are a reason of popular concern as a result of their likely harmful results on human safety [3].

Although human exposure to pesticides occurs through different ways, consumption of agricultural commodities containing pesticides is the major route. Most pesticide residues are appeared in food because of the direct usage of a pesticide to agricultural products or in the period of storage. For example, Lacina et al. reported that organophosphates such as chlorpyrifos-methyl and malathion were principal residues in wheat samples in the storage times [4]. Nowadays, because of food security, application of pesticides is inevitable in the world. Therefore, different countries and international organizations have included various regulations for supporting of human health [5, 6]. Consequently, sound management of pesticide residues in agricultural products, according to national and international regulations require advanced analytical methods.

A good analytical method for detecting pesticide residues in various foods must be able to measure residues at extremely low amounts, and must prepare unequivocal document to establish both the identification and quantitation of residues [7, 8]. In recent decades, various sample preparation techniques have been introduced for quantitation of pesticide residues in food matrices and the most used named QuEChERS (Quick, Easy, Cheap, Effective, Rugged and Safe). This technique supplies different polar, semi-polar and non-polar pesticide in food samples. Sample preparation technique in QuEChERS method includes three major steps. Firstly, blended samples are extracted with acetonitrile, then magnesium sulfate was added for salting-out partitioning. Finally, matrix molecules are removed using primary secondary amine (PSA) sorbent [9]. Nowadays, combination of the mentioned procedure and liquid and gas chromatography coupled mass spectrometry have been successfully used to assess various pesticides in different food samples [10–16].

Tomato (*Lycopersicon esculentum* Mill) is one of the main agricultural products in the world, including Iran. The use of pesticides in order to increase tomato production affects the whole system of tomato. In addition to commonly used pesticides, the presence of banned pesticides in tomato is another important challenge. Therefore, monitoring of the commonly used and forbidden pesticides in tomato crops requires modern techniques.

In this investigation, a validated multi-residue technique was developed for identification and determination of various pesticide residues in tomato, applying GC-MS and QuEChERS method. Thereafter, the validated method was used for detection and quantitation of 81 pesticide residues in 150 tomato samples gathered from various regions of Iran.

## Methods

### Chemicals and reagents

Reference standards of studied pesticides (Table 1), triphenylphosphate (TTP), and anhydrous magnesium sulfate (MgSO_4_), were obtained from Sigma-Aldrich (Germany). Methanol (MeOH) and HPLC-grade acetonitrile (MeCN) were obtained from Acros (Belgium). Ethyl acetate (EtAc), glacial acetic acid (HOAc) and sodium acetate were purchased from Merck (Darmstadt, Germany). Bondesil-primary secondary amine (PSA, 40 μm) was supplied from Interchim (France). HPLC grade water was obtained by purifying demineralized water on a Milli-Q Plus ultra-pure water system (Millipore, Molsheim, France).

**Table 1:** Summary of names, molecular weights, retention times, diagnostic and quantitative ions for the studied pesticides

No.	Compound	Molecular weight (g/mol)	Diagnostic ions	Quantitative ion	Retention time (min)
1	Teflubenzuron	381.11	160, 225, 355	223	6.71
2	Molinate	187.3	187, 83	126	9.21
3	Diphenylamine	169.23	168,167,170	169	10.97
4	Phorate	260.38	121,97,170	75	12.85
5	Thiometon	246.35	125, 89,93	88	13.47
6	Dimethoate	229.26	93	87	13.96
7	Beta-HCH	290.83	219, 183,217	181	14.91
8	Lindane	290.83	183, 219,217	181	15.18
9	Quintozene	295.36	249, 239,217	237	15.50
10	Diazinon	304.35	137, 199	304	16.08
11	Disulfoton	274.4	89	88	16.45
12	Delta-HCH	290.83	219, 183,	181	16.78
13	Chlorothalonil	265.91	264, 268	266	17.23
14	Propanil	218.08	163,217	161	18.69
15	Primicarb	238.29	72,238	166	17.85
16	Vinclozolin	286.11	198, 187,285	212	19.05
17	Chlorpyrifos-methyl	322.5	289, 290, 288	286	19.36
18	Carbaryl	201.22	115,116	144	20.08
19	Alachlor	269.76	188,146	160	19.87
20	Metalaxyl	279.33	132, 160,192	206	20.24
21	Fenitrothion	277.23	277,109	260	21.32
22	Pirimiphos-methyl	305.33	276,305	290	21.43
23	Dichlofluanid	333.23	167,224	123	21.57
24	Malathion	330.35	125,127	173	22.08
25	Aldrin-R	364.91	265	263	22.32
26	Fenthion	278.33	125, 169,109	278	22.74
27	Chlorpyrifos	350.59	199, 314	197	22.88
28	Dicofol	370.48	141	139	23.09
29	Triadimefon	291.73	181,128	208	23.13
30	Cyprodinil	225.29	225	224	24.84
31	Heptachlor epoxide (cis)	389.31	354	353	25.32
32	Penconazole	284.18	248	159	25.45
33	Heptachlor epoxide (trans)	389.31	217	183	25.67
34	Captan	300.59	149,117	79	25.40
35	Fipronil	437.15	369, 351	367	26.29
36	Triadimenol	295.76	168,128	112	26.33
37	Triflumazole	345.08	206, 179, 287	278	27.05
38	Methidathion	302.3	85	145	27.33
39	o,p-DDE	318.02	248, 176	246	27.62
40	Endosulfan-alpha	406.93	237, 339, 265	241	28.10
41	Butachlor	311.84	160	176	28.48
42	Fenamiphos	303.35	154	303	29.18
43	Imazalil	297.18	173, 217	215	29.73
44	Tricyclazole	189.23	162,161	189	29.32
45	Profenphos	373.63	208	139	29.88
46	Pretilachlor	311.85	238, 176, 202	162	30.16
47	p,p-DDE	318.02	318, 316, 248	246	30.15
48	Oxadiazon	345.22	177, 344,302,258	175	30.59
49	Carboxin	235.3	235	143	30.97
50	o,p-DDD	320.04	237,165	235	30.84
51	Buprofezin	305.44	172	105	31.01
52	Endosulfan-beta	406.93	237, 3389, 267	195	32.69
53	p,p-DDD	320.04	237,165	235	33.59
54	o,p-DDT	354.49	237,165	235	33.78
55	Ethion	384.48	97, 153	231	34.07
56	Triazophos	313.31	162,172	161	35.22
57	Benalaxyl	325.4	91	148	35.90
58	Edifenphos	310.37	173, 310	109	36.01
59	Endosulfan-sulfate	406.93	387,389,385	272	36.28
60	Propiconazole I	342.22	259,175	173	36.30
61	Fenhexamide	302.19	177	97	35.54
62	Propiconazole II	342.22	259,175	173	36.79
63	Tebuconazole	307.82	250	125	37.61
64	Triphenylphosphate^*^	326.28	325	326	38.09
65	Iprodione	330.17	316, 187	314	39.58
66	Phosmet	317.32	161, 133,93	160	39.88
67	Bifenthrin	422.87	166, 123	181	40.32
68	Methoxychlor	345.65	228	227	40.48
69	Fenpropathrin	349.43	181, 208	97	40.67
70	Azinphos-methyl	317.32	132, 160	160	42.15
71	Phosalone	367.81	121,154	182	42.15
72	Amitraz	293.41	162,174,121	293	43.05
73	Lambda Cyhalothrin	449.85	197, 208	181	43.36
74	Fenarimol	331.2	219	139	43.58
75	Bitertanol	337.41	168	170	45.03
76	Permethrin I	391.28	163, 183, 184	183	45.24
77	Permethrin II	391.28	163, 183, 184	183	45.54
78	Prochloraz	376.67	310	180	45.94
79	Fenbuconazole	336.82	198	129	46.58
80	Cypermethrin-alpha	416.3	181, 208	163	47.62
81	Esfenvalerate	419.9	167,181	125	49.82
82	Deltamethrin	505.21	253,255	181	51.231

*Internal standard

Individual standard stock solutions (1.0 mg/mL) of the investigated pesticides were prepared at 20 °C by dissolving in EtAc or MeOH. For validation studies, a mixed standard solution (5 μg/mL) was prepared by diluting of the stock standard solutions in MeOH. A stock solution of TTP in ethyl acetate at a concentration of 20 μg/mL was used as internal standard. Some of the investigated pesticides were selected based on chemicals used for tomato production in Iran and Iranian National Standard Organization (INSO) has established MRLs for them. The other pesticides are forbidden to be use in Iran.

### Tomato samples

One hundred and fifty tomato samples produced in different regions of Iran were collected for analysis. In order to avoid possible thermal decomposition of pesticide residues, a 100-g portion of the collected samples was grinded with 100 g dry ice and immediately analyzed.

### Gas chromatography-mass Spectrometry (GC-MS)

Gas chromatography (Model 7890 A, Agilent technologies, USA) with a single quadruple mass Spectrometry detector (Model 5975 C, Agilent technologies, USA) equipped with split/splitless injector and an Agilent auto-sampler with a HP-5 19091S-436 Agilent capillary column (60 m × 0.25 mm I.D., 0.25 μm film thicknesses) was used.

### GC-MS analysis

Helium (99.999%) was employed as the carrier gas at a constant flow rate of 1.6 mL/ min. The oven temperature was programmed from 60 °C (held 1 min), at 30 °C/min to 180 °C, at 2 °C/min ramp to 230 °C, at 5 °C/min ramp to 280 °C, followed by 10 °C/min ramp to 300 °C for 4 min. Injection port was adjusted at 250 °C and splitless mode was employed. After acquiring the ion chromatogram in selected ion monitoring (SIM) mode, peaks were identified by their retention time and mass spectra. The most abundant ion that had the highest signal-to-noise ratio and showed no evidence of chromatographic interference was taken for quantification.

### Sample preparation

Sample preparation was carried out by the original QuEChERS method [9]. Five grams of homogenized tomato sample was weighed into a 50 mL centrifuge tube and 200 ng/g TTP was added as internal standard. Ten mL of acetonitrile (MeCN) was added and the mixture was vigorously shaked for 2.0 min, followed by the addition of a mixture of 2 g anhydrous MgSO_4_ and 1.5 g sodium acetate and vortex mixing for 2.0 min again. The tube was tightly closed and the mixture was centrifuged for 5 min at 9000 rpm. Five mL of supernatant was transferred to a tube containing 60 mg anhydrous MgSO_4_ and 20 mg PSA (primary secondary amine). The mixture was shaked vigorously for 2 min and centrifuged for 5 min at 9000 rpm. Finally, a 0.5 mL of the cleaned supernatant was transferred into a screw cap vial and 1.0 μL of the solution was injected into GC-MS.

### Method validation

According to the European SANCO guidelines [17], the validation study was tested to assess for linearity, recovery, precision, and limits of detection (LOD) and quantitation (LOQ). The linearity of the method was studied applying matrix-matched calibrations by analyzing six concentration levels, between 20 and 200 ng/g. For determination of mean recoveries (to estimate the accuracy of the method) and precision (repeatability, expressed as coefficient of variation in %), five spiked blank tomato samples at concentration levels of 25, 50 and 100 ng/g were prepared and then treated according to the procedure earlier described in sample preparation. Limits of LOD and LOQ were calculated to be the concentrations of pesticide that result in a signal-to-noise ratio of 3 and 10, respectively.

### Quantitation of pesticide residues

The amounts of pesticide residues in the positive samples were calculated by comparing signal from each pesticide with signal from internal standard in the sample on the matrix-matched calibration curve. In order to correct for the losses of pesticide residues during sample preparation, internal standard (TPP) was employed. Excel software was used for statistical calculations.

#### Data availability

The authors do not wish to share their data. All the necessary data have been mentioned in the paper. Please contact corresponding author for data requests.

## Results

### GC-MS determination

For analysis of the studied pesticides, the SIM mode was applied. Quantitation and confirmation of pesticides were performed based on the use of: one quantitative ion, at least one diagnostic (or qualifier) ion, and retention times. Table 1 summarizes molar weights, retention time, and SIM parameters obtained for the studied pesticides.

### Method validation

The investigated method was validated by determining the limits of detection (LOD) and quantitation (LOQ), the recovery and precision at different levels of the fortification. The method validation revealed that the calibration curves for each analyte were linear in the concentration range of 20–200 ng/g with a determination coefficient (R^2^) in the range between 0.993 and 0.999. As shown in Table 2, the LODs and LOQs were in the range between 2.5–6.7 ng/g and 7.5–20 ng/g respectively. The mean recoveries obtained for three fortification levels (25, 50 and 100 ng/g -five replicates each) were 72–116% with satisfactory precision (RSD < 20%), meeting EU guidelines method performance criteria [17].

**Table 2:** Mean recoveries (%), relative standard deviations (RSD, %), LOQs and LODs (ng/g) obtained for studied pesticides in tomato samples, spiked at 25, 50 and 100 ng/g levels (*n* = 5)

No.	Compounds	25 ng/g		50 ng/g		100 ng/g		LOQ^a^	LOD^b^
		Mean	RSD	Mean	RSD	Mean	RSD		
1	Teflubenzuron	101	12	105	6	95	10	16.0	5.3
2	Molinate	103	14	79	9	93	11	9.5	3.2
3	Diphenylamine	115	8	86	13	107	5	10.5	3.5
4	Phorate	92	10	94	8	93	12	15.5	5.2
5	Thiometon	89	7	86	11	91	3	20.0	6.7
6	Dimethoate	98	9	97	9	80	7	16.5	5.5
7	Beta-HCH	92	16	106	4	89	7	9.0	3.0
8	Lindane	114	11	109	9	83	6	10.5	3.5
9	Quintozene	96	4	103	5	85	6	17.0	5.7
10	Diazinon	90	15	95	12	100	9	16.0	5.3
11	Disulfoton	95	6	95	4	85	9	11.5	3.8
12	Delta-HCH	86	13	99	9	100	6	13.0	4.3
13	Chlorothalonil	92	15	104	6	89	8	14.5	4.8
14	Propanil	75	10	83	6	77	7	15.0	5.0
15	Primicarb	85	5	98	8	95	11	11.0	3.7
16	Vinclozolin	83	12	99	4	97	4	14.0	4.7
17	Chlorpyrifos-methyl	111	2	84	6	86	2	19.0	6.3
18	Carbaryl	109	5	98	7	89	3	20.0	6.7
19	Alachlor	96	9	80	3	100	2	16.5	5.5
20	Metalaxyl	90	12	85	8	97	2	11.5	3.8
21	Fenitrothion	108	9	105	7	95	3	9.0	3.0
22	Pirimiphos-methyl	89	1	102	6	88	6	7.5	2.5
23	Dichlofluanid	108	8	92	12	111	5	9.5	3.2
24	Malathion	98	4	81	7	100	2	13.5	4.5
25	Aldrin-R	101	13	94	6	99	7	18.0	6.0
26	Fenthion	85	12	93	16	103	9	19.5	6.5
27	Chlorpyrifos	106	13	93	11	97	6	13.0	4.3
28	Dicofol	112	5	89	9	100	7	14.0	4.7
29	Triadimefon	95	3	88	1	89	6	15.0	5.0
30	Cyprodinil	102	7	98	12	95	11	17.0	5.7
31	Heptachlor epoxide (cis)	102	8	85	9	99	2	8.0	2.7
32	Penconazole	82	9	88	5	109	10	9.5	3.2
33	Heptachlor epoxide (trans)	80	5	96	2	100	1	10.0	3.3
34	Captan	113	7	82	6	106	2	12.0	4.0
35	Fipronil	95	5	98	2	112	8	17.5	5.8
36	Triadimenol	107	4	100	6	83	4	18.0	6.0
37	Triflumazole	96	7	85	12	110	4	20.0	6.7
38	Methidathion	88	11	87	8	109	6	14.0	4.7
39	o,p-DDE	86	9	88	16	87	10	10.0	3.3
40	Endosulfan-alpha	72	8	86	13	107	9	11.0	3.7
41	Butachlor	102	6	86	14	105	8	14.5	4.8
42	Fenamiphos	109	14	88	16	97	9	17.0	5.7
43	Imazalil	86	11	96	9	100	3	16.5	5.5
44	Tricyclazole	103	2	97	7	90	6	17.0	5.7
45	Profenphos	101	5	90	11	110	9	18.5	6.2
46	Pretilachlor	89	9	86	6	95	9	17.0	5.7
47	p,p-DDE	112	6	87	8	108	3	17.5	5.8
48	Oxadiazon	95	7	88	12	106	6	13.5	4.5
49	Carboxin	80	9	78	11	98	9	12.5	4.2
50	o,p-DDD	88	12	95	6	107	11	10.5	3.5
51	Buprofezin	98	11	89	6	104	10	12.5	4.2
52	Endosulfan-beta	79	6	96	12	80	10	8.0	2.7
53	p,p-DDD	81	8	94	3	110	2	13.0	4.3
54	o,p-DDT	109	2	93	5	87	3	18.0	6.0
55	Ethion	101	9	89	5	113	9	17.0	5.7
56	Triazophos	97	8	110	9	83	13	15.5	5.2
57	Benalaxyl	105	6	98	3	108	5	15.0	5.0
58	Edifenphos	100	9	111	7	114	6	19.5	6.5
59	Endosulfan-sulfate	98	12	98	16	105	10	19.0	6.3
60	Propiconazole I	90	7	88	10	103	9	17.0	5.7
61	Fenhexamide	95	9	101	3	111	6	19.0	6.3
62	Propiconazole II	113	15	88	11	78	15	18.0	6.0
63	Tebuconazole	87	11	92	4	100	13	15.0	5.0
64	Iprodione	97	9	87	2	98	9	8.0	2.7
65	Phosmet	107	17	99	10	112	2	18.5	6.2
66	Bifenthrin	116	6	100	11	96	6	13.0	4.3
67	Methoxychlor	100	2	89	8	77	3	14.5	4.8
68	Fenpropathrin	100	7	98	2	90	1	13.5	4.5
69	Azinphos-methyl	85	3	99	7	94	1	19.0	6.3
70	Phosalone	108	5	102	2	86	7	15.0	5.0
71	Amitraz	100	11	84	7	91	8	19.0	6.3
72	Lambda Cyhalothrin	89	8	102	2	105	3	19.5	6.5
73	Fenarimol	84	6	112	14	98	10	19.5	6.5
74	Bitertanol	99	3	106	11	111	10	13.5	4.5
75	Permethrin I	114	7	97	6	87	3	9.5	3.2
76	Permethrin II	105	6	89	3	112	5	10.0	3.3
77	Prochloraz	84	9	108	10	103	4	19.5	6.5
78	Fenbuconazole	87	4	97	2	96	3	20.0	6.7
79	Cypermethrin-alpha	88	8	96	6	88	10	20.0	6.7
80	Esfenvalerate	89	4	97	11	84	9	11.5	3.8
81	Deltamethrin	89	13	101	5	100	9	10.5	3.5

^a^Limit of quantitation

^b^Limit of detection

### Analysis of real samples

The validated method was applied for analysis of 150 tomato samples collected from different regions of Iran. As shown in Table 3, six pesticides including; iprodione, permethrine (sum of permethrine I and II), esfenvalerate, chlorpyrifos, diazinon and penconazole were detected in 31 (20.7%) samples. Among the detected pesticides, iprodione was the most common pesticide residues (6.0%), followed by (4.7%), permethrine (4.7%), esfenvalerate (4.7%), chlorpyrifos (3.3%), diazinon (2.0%) and penconazole (1.3%).

**Table 3:** Pesticide residues determined in tomato samples collected from different regions of Iran

No.	Pesticides	No. of positive samples	LOD^a^ (ng/g)	LOQ^b^ (ng/g)	Min level (ng/g)	Max level (ng/g)	No. of positive samples>MRL	INSOʾs^c^ MRL (ng/g)
1	Iprodione	9(6.0%)	2.7	8.0	14	359	–	5000
2	Permethrine^d^	7(4.7%)	3.2	9.5	44	347	–	1000
3	Esfenvalerate^e^	7(4.7%)	3.8	11.5	13	51	–	–
4	Chlorpyrifos	5(3.3%)	4.3	13	24	219	2(40%)	100
5	Diazinon	3(2.0%)	5.3	16	38	232	2(66.7%)	50
6	Penconazole^e^	2(1.3%)	3.2	9.5	92	102	–	–

^a^Limit of Detection

^b^Limit of Quantitation

^c^Iranian National Standard Organization

^d^Sum of permethrine I and II

^e^Prohibited pesticides for tomato production in Iran

## Discussion

Tomato (*Lycopersicon esculentum* Mill.) is one of the most important vegetables in the world. Tomato contains nutrients such as vitamin A, vitamin C, potassium, phosphorus, magnesium, and calcium [18]. It also contains lycopene, an antioxidant that reduces the risk of cancer [19]. World and Iran tomato production in 2015 were more than 163 and 6 million tons of fresh fruit, respectively [20]. During tomato production, different insects and mites attack different parts of tomato. Therefore, prevention and control of pests in tomato is very important. In Iran, different classes of pesticides are registered to control of pests in tomato. However, most pesticide residues can remain in tomato and affect human health.

According to the Iranian regulations, the studied pesticides can be divided into three groups: 1) forbidden or banned pesticides, 2) registered and, 3) not registered pesticides for tomato production in Iran. Group 1, including, azinphos-methyl, aldrin, delta- HCH, phorate, lindane, methidathion, methoxychlor, triazophos and DDT are forbidden for crop production, including tomato in Iran. These pesticides severely affected human health and, their chronic toxicity has been documented. For example, it has been shown that phorate, an organophosphorus pesticide, causes genotoxicity [21] and leading to prostate cancer [22]. Therefore, it is necessary to detect banned pesticides in tomato. The results showed that none of the detected pesticide was forbidden However, Bakore et al. detected organochlorine residues of DDT, HCH and aldrin in all of the studied tomato samples in India [23].

Group 2, including deltamethrin, permethrine, diazinon, chlorpyrifos, malathion, carbaryl, fenitrothion, fenthion, captan, iprodione, tebuconazole, propiconazole, carboxin and dimethoate are registered for tomato production in Iran and the MRLs for them have been established by the Iranian National Standard Organization. As shown in Table 3, among the registered pesticides, only iprodione, permethrine, chlorpyrifos and diazinon found in positive tomato samples. With exception of Chlorpyrifos and diazinon the concentrations of iprodione and permethrine were found below the INSO-specified MRLs. Chlorpyrifos and diazinon are effective organophosphate chemicals applied largely across the world in agricultural and domestic pest control. They have three major ways of toxicity in animals: blockage of the acetylcholine esterase enzyme (AChE), oxidative damage, and interruption of endocrine systems. Inhibition of AChE causes over-stimulation of related neurons in the CNS, resulting in sensorial and behavioral disturbances, general weakness, increased secretions such as urination, salivation and lacrimation, depression of motor function and respiration, ataxia, tremor, convulsions, coma and death [24]. Several investigations have shown that chlorpyrifos causes oxidative damage in animals. Oxidative stress and AChE inhibition, underlies the prenatal neurotoxicity of chlorpyrifos [25]. Furthermore, oxidative stress induces dopaminergic damages in the central nervous system may lead to Parkinson’s disease [26]. Chlorpyrifos can strongly block CYP450 enzymes in the liver and, long exposure can lead to the liver hurt and various metabolic disorders [27]. Chronic toxicity of chlorpyrifos can impair kidney structure that may lead to renal failure [28]. In addition, a number of investigations show that chlorpyrifos may be lead to lung and rectal cancers in humans [29].

The other pesticides in Table 1 belong to group 3. These chemicals are allowed to be used in other crop production, like apple, cucumber, rice etc., but are not registered for tomato production, and MRLs have not been established for them by INSO. Among the detected pesticides, esfenvalerate and penconazole belong to this group and their occurrence in tomato samples is a major concern. Additionally, some tomato samples included more than one pesticide; the reason being that tomato cultivated under some conditions is highly sensitive to pests and requires successive applications of different pesticide treatments.

## Conclusions

In the present study, an accurate, precise, sensitive and selective method was developed for the simultaneous detection, quantification and confirmation of 81 pesticide residues (belonging to different chemical families) in tomato employing QuEChERS sample preparation procedure and GC-MS. The validated results showed excellent recoveries (71–119%) and precision (RSDs <20%) for all studied pesticides, meeting EU guidelines method performance criteria. The method was applied successfully for the analyses of 150 tomato samples collected from different market of Tehran. Six compounds were found in 31 positive samples. Iprodione was the most common detected residues, followed by permethrine, esfenvalerate, chlorpyrifos, diazinon, and penconazole. Chronically, the detected pesticides in tomato can affect Iranian consumers in long time. Therefore, it is necessary to control and management of their residues in tomato by applying Good Agricultural Practice (GAP) and implementing integrated pest management (IPM) in Iran.

## Abbreviations

*AChE*: acetylcholine esterase
*CNS*: central nervous system
*EtAC*: ethyl acetate
*EU*: european union
*GAP*: good agricultural practice
*GC-MS*: gas chromatography-mass spectrometry
*HOAc*: acetic acid
*INSO*: iranian national standard organization
*IPM*: integrated pest management
*LC-MS*: liquid chromatography-mass spectrometry
*LOD*: limit of detection
*LOQ*: limit quantitation
*MeCN*: acetonitrile
*MeOH*: methanol
*MRL*: maximum residue limit
*PSA*: primary secondary amine
*QuEChERS*: quick, easy, cheap, effective, rugged and safe
*TTP*: triphenylphosphate
